# Myopia management -A survey of optometrists and ophthalmologists in Israel

**DOI:** 10.1177/11206721231211465

**Published:** 2023-10-30

**Authors:** Nir Erdinest, Naomi London, Yair Morad, Shehzad A Naroo

**Affiliations:** 1 Department of Ophthalmology, Hadassah-Hebrew University Medical Center, Faculty of Medicine, Hebrew University of Jerusalem, Jerusalem, Israel; 2 Private practice, Jerusalem, Israel; 3 Department of Ophthalmology, 37256 Shamir Medical Center, Tzrifin, Israel; 4 College of Health and Life Sciences, Aston University, Birmingham, UK

**Keywords:** myopia management, myopia control, eye care practitioners, optometrists, ophthalmologists, survey

## Abstract

**Purpose:**

Myopia management is practiced by ophthalmologists and optometrists**.** This study evaluated the approach and standard of myopia management among eye-care practitioners (ECPs) in Israel. The findings may ultimately affect the quality of care.

**Methods:**

A questionnaire was sent to 954 optometrists and 365 ophthalmologists, including demographic questions; whether they owned any devices to monitor myopia progression; the lowest progression they considered significant; various questions pertaining to myopia management and treatment methods.

**Results:**

Responses from 135 optometrists and 126 ophthalmologists were collected, the majority practicing more than five years; 94% of optometrists, and 64% of ophthalmologists. Around 53% of optometrists and 27% of the ophthalmologists proclaimed to practice myopia management. ECPs primary parameters influencing risk assessment for progression were age, genetic background and history of progression. Time outdoors, during daylight hours, is advised by ophthalmologists (97%) and optometrists (78%). Limiting screentime is encouraged by 87% of ophthalmologists and 69% of optometrists. Myopia progression of 0.50D–0.75D after six months is regarded to require intervention by 93% of ophthalmologists and 83% of optometrists. Optometrists selected multiple myopia management treatments, primarily optical (ophthalmic myopia management lenses 40%, multifocal ophthalmic lenses 24%, peripheral blur contact lenses 38%, orthokeratology 11%), while 95% of ophthalmologists chose atropine and only 3–11% selected any additional treatments to consider.

**Conclusion:**

This study highlighted ECPs’ agreement on the principles, importance of, and timeline of intervention with myopia management. The disconnect between the two professions lies in management methods. Genuine dialogue and co-management should be encouraged for maximum implementation, benefit and effectiveness of available patient treatments.

## Introduction

With its concomitant physiological pathologies and economic burden, the increasing prevalence of myopia has stimulated the development of multiple management options. Research is actively underway in the realm of understanding the exact mechanism of myopia progression from a biological, genetic, biochemical and environmental perspective. The continuous search for the ultimate method to control myopia is just as broad, ranging from topical and oral pharmaceuticals to optical and environmental measures.^1,2^

Israel is a first-world country with commendable technology industry and boasts an advanced healthcare system, considered one of the world's highest-quality healthcare systems. Healthcare providers are educated nationally or from diverse international backgrounds and are licensed after taking national Board of Health examinations. Eye care is provided both by ophthalmologists and optometrists, where the responsibilities are roughly divided into ocular health and vision correction respectively, with some overlap and co-management between the two professions. Based on the Israel Ministry of Health data from October 2022, 2750 optometrists^3^ and 1006 ophthalmologists^4^ are nationally licensed. Although eye care practitioners (ECPs) have access to global frontline research and data, and indeed many are involved in myopia management research, it is unclear what the prevalence of awareness and implementation of the various locally available options to the population is. This survey will help to understand the current myopia management strategies, perceived effectiveness and implementation.

## Methods

A Hebrew language questionnaire comprised of a total of eleven items was sent from July to September 2022, with the assistance of the Israel Council on Optometry (ICO) and the Israel Ophthalmological Society (IOS) via E-mail or WhatsApp message (WhatsApp LLC, 1601 Willow Road, Menlo Park, California USA), to 1319 ECPs (consisting of 954 optometrists and 365 ophthalmologists) (Table 1). Responses were collected anonymously through an online survey using a Google Forms platform (Google Inc., 1600 Amphitheatre Parkway Mountain View, CA 94043, USA). Institutional ethical approval was sought, but the study was exempted since no personal information was collected. Nonetheless, informed consent was obtained from each participant, and a participant information sheet was available. Participants were also advised that participation was voluntary, and their survey completion confirmed their consent.

**Table 1.** Survey distribution characteristics.

Eye care practitioners	Distribution	Responded to the survey	
		n	Response rate
Optometrists	954	135	14.15%
Ophthalmologists	365	126	34.52%
Total	1319	261	20%

Demographic questions asked whether the ECP was an ophthalmologist or an optometrist, an employee or self-employed and whether they had been practicing for more or less than five years. The survey enquired if the ECP had any devices specifically designed to monitor myopia progression, such as a biometer. They were asked whether they practice myopia management and, if the answer was negative, to select the primary reason for not implementing it from a list of choices.

The following questions pertained to the individual’s reasoning and personal implementation of myopia management, including, in their opinion, what the lowest progression over six months to warrant beginning treatment. The three following questions provided the opportunity to select multiple options. These questions were: If not providing specialized treatments, what conventional treatments do they recommend to their patients? What types of treatments do they implement and support? What data do they rely on when implementing myopia management treatments?

The final question was open-ended, allowing the ECP to comment on the survey. Appendix 1 is a translated version of the survey.

## Results

Two hundred and sixty-one ECPs responded, 52% were optometrists, and 48% were ophthalmologists. Response rates are given in Table 1. The ECPs who were exclusively employees comprised 67% (n  =  176) of the responders (135 of those were optometrists, 41 were ophthalmologists). A total of 28% (n  =  73; 58 ophthalmologists and 15 optometrists) were in independent practice. Around 5% of the ECPs identified as independent and also employees, meaning they work both as independents (either as freelancers or own a private practice) and are also employed by another institution or clinic.

**Figure 1.**
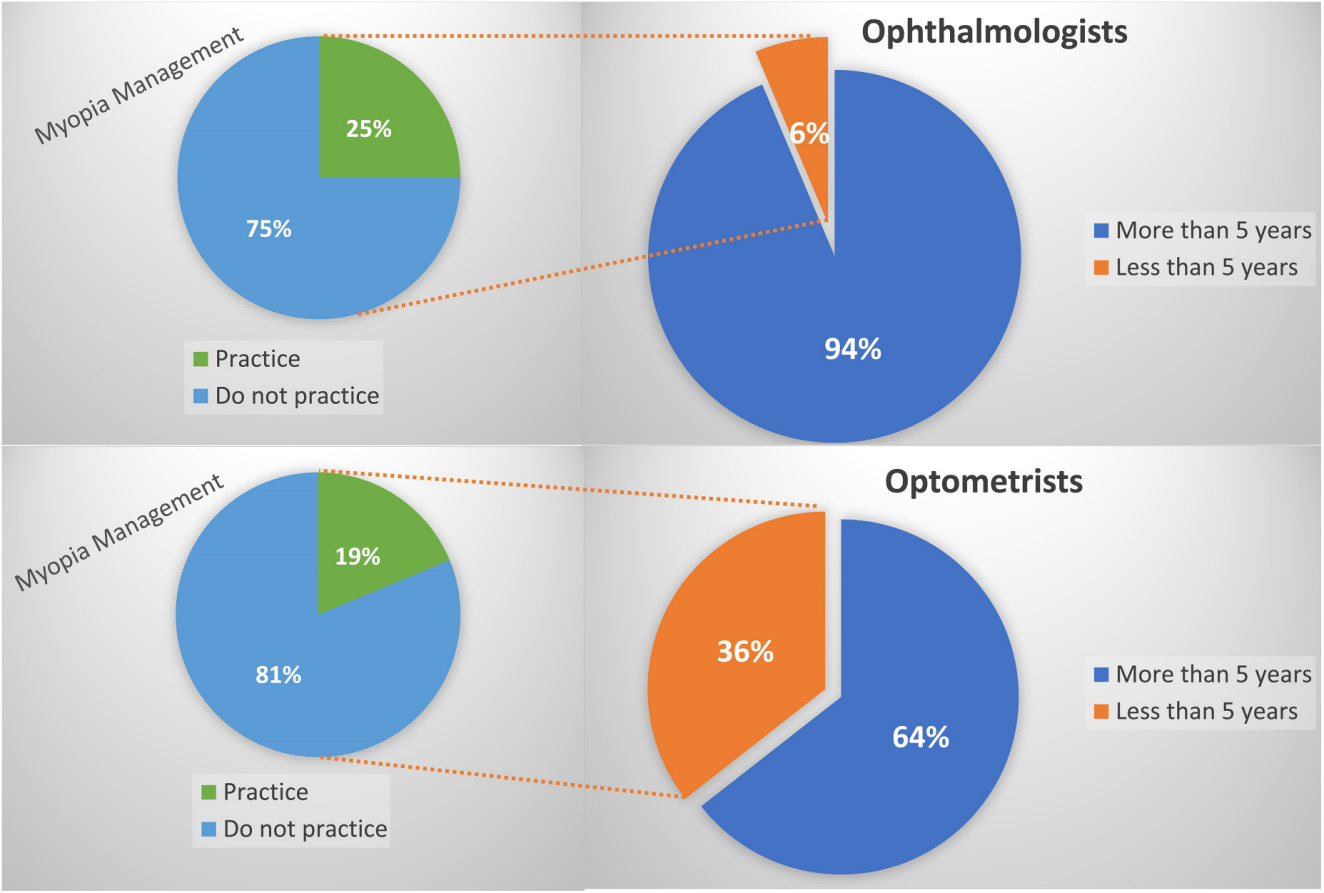
ECPs with more or less than five years of experience that practice myopia management.

While 53% of the optometrists claimed to practice myopia management, only 27% of the ophthalmologists did. The principal reason cited for not practicing myopia management by both optometrists (20%) and ophthalmologists (19%) was the high price of treatments combined with the inability to guarantee success (Figure 1).

Only 2% (n  =  3) of optometrists own designated imaging instrumentation for myopia management, while 19% (n  =  24) of ophthalmologists responded that they had this type of equipment.

**Figure 2.**
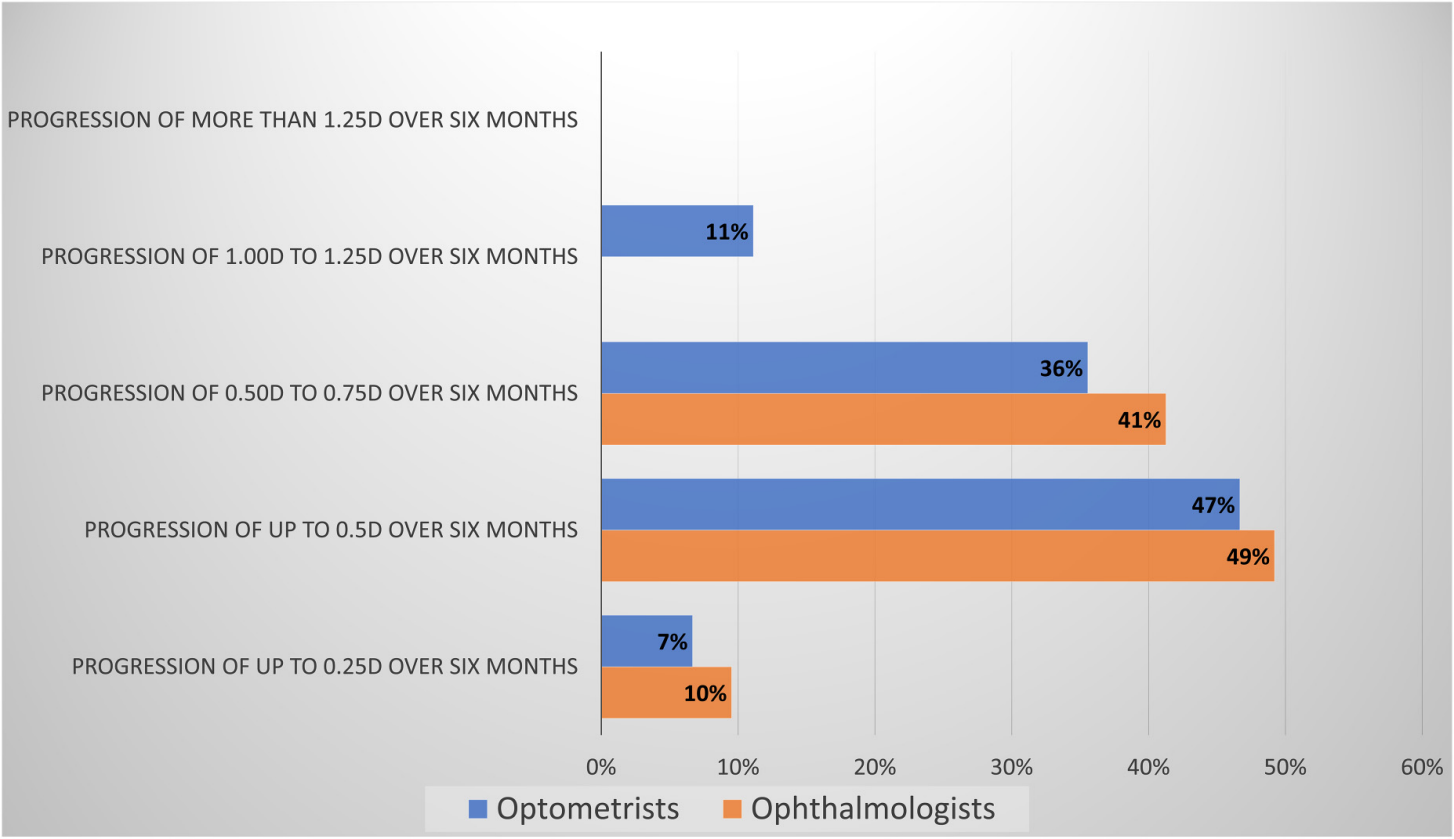
ECPs consideration of most moderate myopia progression requiring intervention.

The lowest myopia progression over six months selected as requiring intervention was considered 0.50 to 0.75D by 90% of the ophthalmologists and 83% of the optometrists (Figure 2).

**Figure 3.**
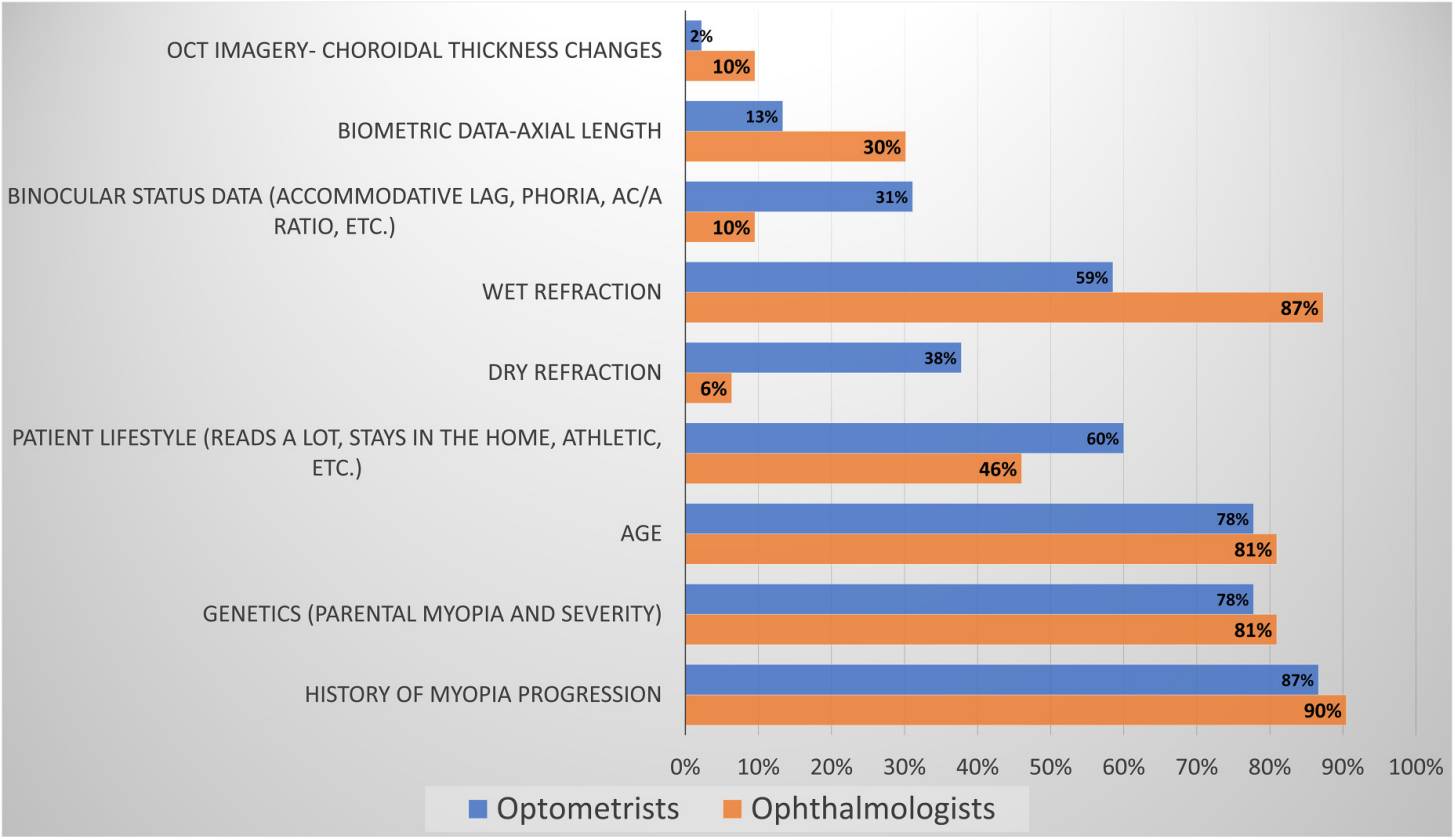
Parameters influencing ECPs management implementation.

The primary parameters influencing the risk assessment of a patient's myopia progression, that was almost equal between ophthalmologists and optometrists, included the age of the patient (81% and 78%, respectively), genetic background (81% and 78%, respectively) and history of the patient's myopia progression (90% and 87%, respectively). Ophthalmologists favoured cycloplegic refraction (87%) over optometrists (59%). Biometric axial length data influenced the decision process of 30% of the ophthalmologists, and 13% of the optometrists, while binocular status influenced 10% of the ophthalmologists and 30% of the optometrists. Figure 3 presents the complete responses.

**Figure 4.**
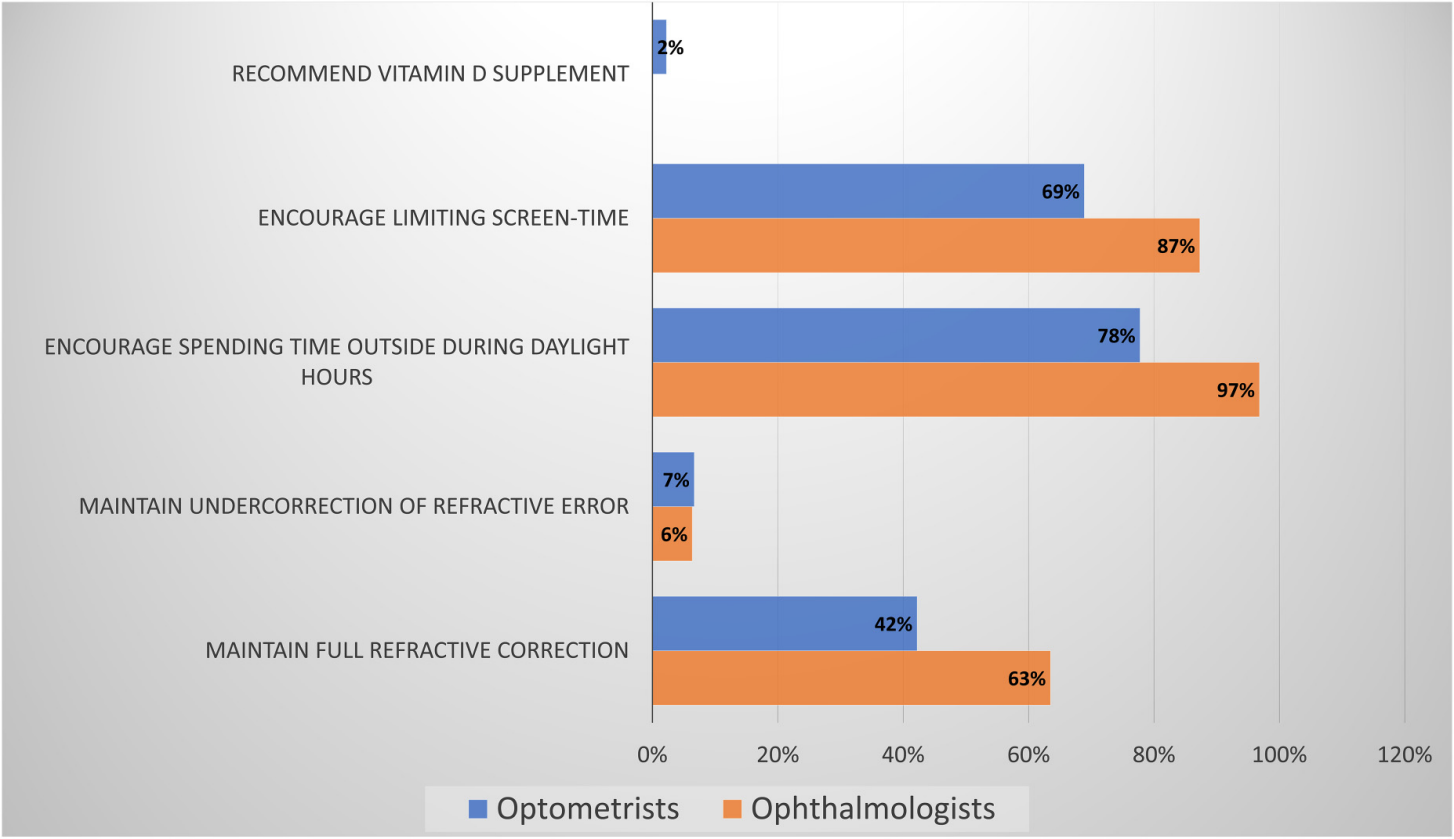
ECPs conventional treatments recommendations.

ECPs were asked what recommendations, not considered direct myopia treatment, they advised to their patients. The importance of spending time outdoors during daylight hours is advised by 97% of responding ophthalmologists and 78% of optometrists. Limiting screentime is also encouraged by 87% of ophthalmologists and 69% of responding optometrists. None of the ophthalmologists and only 2% of the optometrists address vitamin D supplementation with their patients. Complete responses are detailed in Figure 4.

**Figure 5.**
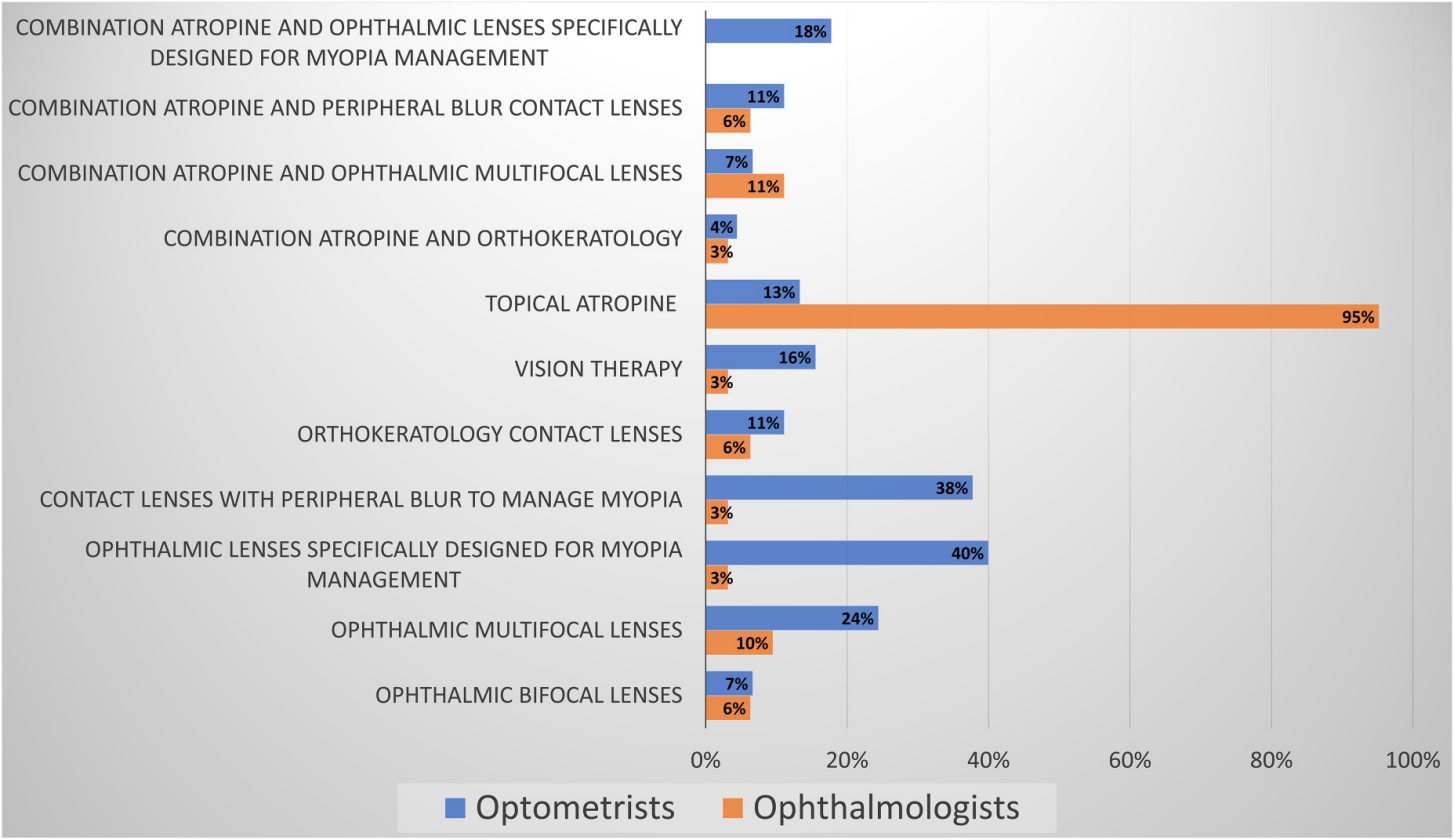
Myopia management treatments that ECPs support.

Optometrists selected multiple myopia management treatment options, while 95% of ophthalmologists selected atropine as their primary treatment choice, and only 3–11% selected additional treatments (Figure 5).

## Discussion

This survey presents the current paradigm of myopia management for ECPs in Israel. It shows that although more optometrists are inclined towards and practice myopia management than ophthalmologists, the ownership of designated equipment lies more with the ophthalmologists. Most of the optometrists that responded (88%, n  =  120) were employees, so investing in expensive equipment with no government or health insurance reimbursement is rare and purchasing equipment may also be a decision they do not control.

Interestingly, all ECPs seem to agree that 0.50–0.75D is the minimum amount of myopia progression over six months where intervention is appropriate and rely on similar additional data such as family history, the child’s habits and hobbies. Ophthalmologists put more emphasis on objective data, such as biometric data (30%) versus optometrists (13%) or cycloplegic refraction (87%) than optometrists (59%). Perhaps this is partly since in Israel diagnostic drugs are not legally accessible to optometrists and many will not have biometric equipment. Perhaps optometrists feel confident with the data acquired from their binocular and accommodative status examinations are reliable enough, together with other parameters, to decide when to intervene.^5–7^

Regarding preventative recommendations, both ophthalmologists and optometrists tended to agree on the importance of providing full distance refraction, the importance of spending time outside in the sunlight and limiting visual display screen time (Figure 4).

However, there is a significant difference in the types of treatments ECPs support. Virtually all responding ophthalmologists (95%) have overwhelming confidence in the science behind topical atropine therapy, while only 13% of optometrists support atropine therapy alone. The most significant differences between the professions lie in increased optometric support for treatment without atropine (7%–40%), particularly specifically designed peripherally defocused ophthalmic lenses (40%) and soft contact lenses (38%). The discrepancy continues with minimal support (3%) by ophthalmologists for vision therapy, whereas 16% of optometrists found it a valuable tool for myopia management. Part of this can be attributed to the access of each profession to the respective treatments. Optometrists cannot prescribe atropine, and ophthalmologists have limited to no experience in contact lens practice or vision therapy.

The disadvantage that conceivably results from this data over the divide observed in practice between the two professions, is the implementation and benefit of the potential effectivity of choosing the possibly most effective treatment for specific patients,^8,9^ or combination treatments which some emerging studies show can be the method of choice in some challenging cases.^10^ Only 3–7% of ophthalmologists and 4–18% of optometrists supported combination treatments such as atropine in addition to orthokeratology, peripheral defocus contact lenses, or ophthalmic spectacle lenses (Figure 5).

A limitation of this survey is the disparity between the percentage of responders in each category. While a similar number of ophthalmologists and optometrists responded to this survey, they represented 12.6% of the nationally licensed ophthalmologists and only 4.9% of the licensed optometrists in the country. Nevertheless, a recently published survey attempting to discover the global trends in myopia management, which reached out to 13 countries’ ECPs, included only 42 responses from Israel, without specifying whether they were an optometrist, ophthalmologist or optician.^11^That study reported lesser perceived effectiveness of under-correction, single vision spectacles and contact lenses, and rigid contact lenses, but did not elaborate on the treatments considered more effective.^11^ They did mention that the Israel responders reported they prescribed more multifocal or myopia control contact lenses and fewer bifocal lens spectacles and combination therapies. This corresponds with the data received from optometrists in the present study. They further suggested a low concern level in Israel regarding myopia in general. However, the current survey points more to a lack of confidence in the outcome rather than a lack of appreciation regarding the need for treatment. An additional limitation is that some of the survey items restricted the number of response options, though not on the questions pertaining to myopia recommendations and preferred treatment options, which is where the ECPs principles become apparent.

Many countries around the globe have both optometrists and ophthalmologists, each with a different balance of responsibilities and collaboration. The importance of this survey lies in the ability to exhibit the similarities in philosophy and belief in the importance of myopia management, yet the great divide between the two primary ocular caregivers to children and teenagers on how to manage them. Each profession seems focused on its own terrain without lateral gaze. The goal in every country should be to strive to work together to create greater dialogue, education, cross-referencing of both published scientific data and anecdotal clinical experience, and genuine co-management. This will benefit the primary goal of all ECPs, the patients’ best interest.

## Supplemental Material

sj-docx-1-ejo-10.1177_11206721231211465 - Supplemental material for Myopia management -A survey of optometrists and ophthalmologists in Israel Supplemental material, sj-docx-1-ejo-10.1177_11206721231211465 for Myopia management -A survey of optometrists and ophthalmologists in Israel by Nir Erdinest, Naomi London, Yair Morad and Shehzad A Naroo in European Journal of Ophthalmology
