# Supplementary material for: Myopia management -A survey of optometrists and ophthalmologists in Israel
Source: Eur J Ophthalmol. 2023 Oct 30;34(4):980–5. doi: 10.1177/11206721231211465 (PMC11295405; doi:10.1177/11206721231211465)
Supplement: sj-docx-1-ejo-10.1177_11206721231211465 - Supplemental material for Myopia management -A survey of optometrists and ophthalmologists in Israel [file sj-docx-1-ejo-10.1177_11206721231211465.docx]

**Supplement 1. Translated version of the survey**

| Myopia management survey Hello, attached is the survey to control myopia that will be published in a leading international journal on the current prevalent approaches in Israel to manage myopia  1. **I am (mark one answer):**   Optometrist / Ophthalmologist   1. **I am (mark one answer):**   Independent (clinic/chain/shop) / Employee (Hospital/Chain/shop)   1. **I have had a Ministry of Health license to practice optometry/ophthalmology for (mark one answer):**   Less than 5 years/ More than 5 years   1. **Do you practice myopia management? (mark one answer):**   Yes / No  **4A. If you are not practicing myopia management, the primary reason for this is (mark one answer):**   1. I avoid testing children and/or teenagers 2. Requires prolonged chair time 3. I do not believe in or support the field - there is not enough professional literature or clinical findings supporting this field 4. The price of treatments for the patients prevents me from treating them, plus the lack of guarantee of success 5. Poor cooperation with colleagues 6. Poor cooperation and lack of communication with parents 7. **Do you have any designated imaging device for myopia management (mark or write one answer):** 8. Yes, a biometer 9. Yes, an integrated device dedicated to myopia management 10. No 11. Other 12. **What, in your opinion, is considered the mildest increase in myopia that requires treatment (mark one answer):** 13. An increase of up to 0.25 diopters over a 6 month period 14. An increase of up to 0.50 diopters over a 6 month period 15. An increase of 0.50 diopters to 0.75 diopters over a 6 month period 16. An increase of 1.00 diopters to 1.25 diopters over a 6 month period 17. An increase of over 1.25 diopters over a 6 month period 18. **A conservative/traditional treatment I recommend (one or more options can be chosen):** 19. Full refraction 20. Recommend the child and/or teenager spends time outdoors during daylight hours 21. Recommendation to limit digital screen time 22. Vitamin D supplement 23. **Myopia management therapy I support/condone (one or more options can be chosen):** 24. Bifocal spectaclelenses 25. Multifocal spectacle lenses 26. Special spectacle lenses to control myopia 27. Contact lenses to prevent peripheral blurring 28. Ortho K contact lenses 29. Visual therapy 30. Topical atropine 31. Combined treatment of atropine and ortho K 32. Combined treatment of atropine and multifocal glasses 33. Combined treatment of atropine and contact lenses to prevent peripheral blurring 34. Combined treatment of atropine and special/dedicated spectacle lenses to control myopia |
| --- |
